# Fluid resuscitation and outcomes in heart failure patients with severe sepsis or septic shock: A retrospective case-control study

**DOI:** 10.1371/journal.pone.0256368

**Published:** 2021-08-19

**Authors:** Roshan Acharya, Aakash Patel, Evan Schultz, Michael Bourgeois, Natalie Kandinata, Rishi Paswan, Smita Kafle, Yub Raj Sedhai, Usman Younus

**Affiliations:** 1 Department of Internal Medicine, Cape Fear Valley Medical Center, Fayetteville, NC, United States of America; 2 RN-BSN Program, Fayetteville State University, Fayetteville, NC, United States of America; 3 Department of Internal Medicine, Virginia Commonwealth University School of Medicine, Richmond, VA, United States of America; 4 Department of Critical Care Medicine, Cape Fear Valley Medical Center, Fayetteville, NC, United States of America; Wayne State University, UNITED STATES

## Abstract

**Background:**

The use of ≥30 mL/Kg fluid bolus in congestive heart failure (CHF) patients presenting with severe sepsis or septic shock remained controversial due to the paucity of data.

**Methods:**

The retrospective case-control study included 671 adult patients who presented to the emergency department of a tertiary care hospital from January 01, 2017 to December 31, 2019 with severe sepsis or septic shock. Patients were categorized into the CHF group and the non-CHF group. The primary outcome was to evaluate the compliance with ≥30 mL/Kg fluid bolus within 6 hours of presentation. The comparison of baseline characteristics and secondary outcomes were done between the groups who received ≥30 mL/Kg fluid bolus. For the subgroup analysis of the CHF group, it was divided based on if they received ≥30 mL/Kg fluid bolus or not, and comparison was done for baseline characteristics and secondary outcomes. Univariate and multivariable analyses were performed to explore the differences between the groups for in-hospital mortality and mechanical ventilation.

**Results:**

The use of ≥30 mL/Kg fluid bolus was low in both the CHF and non-CHF groups [39% vs. 66% (p<0.05)]. Mortality was higher in the CHF group [33% vs 18% (p<0.05)]. Multivariable analysis revealed that the use of ≥30 mL/Kg fluid bolus decreased the chances of mortality by 12% [OR 0.88, 95% CI 0.82–0.95 (p<0.05)]. The use of ≥30 mL/Kg fluid bolus did not increase the odds of mechanical ventilation [OR 0.99, 95% CI 0.93–1.05 (p = 0.78)]. In subgroup analysis, the use of ≥30 mL/Kg fluid bolus decreased the chances of mortality by 5% [OR 0.95, 95% CI 0.90–0.99, (p<0.05)] and did not increase the odds of mechanical ventilation. The presence of the low ejection fraction did not influence the chance of getting fluid bolus.

**Conclusion:**

The use of ≥30 mL/Kg fluid bolus seems to confer protection against in-hospital mortality and is not associated with increased chances of mechanical ventilation in heart failure patients presenting with severe sepsis or septic shock.

## 1. Introduction

More than fifty percent of the patients with sepsis, severe sepsis (SeS), or septic shock (SS) used to die before the widespread implementation of early goal-directed therapy (EGDT) recommended by surviving sepsis campaign (SSC) in 2001 [1–3]. The three large multinational trials ProCESS, ARISE, and ProMISe (trios-trial), did not show substantial mortality benefit of EGDT versus usual care [4]. The patient-level meta-analysis of three trials done also confirmed the finding, but the amount of fluid used in both EGDT and usual care patients was around 28 mL/Kg (~2L) before the patients were randomized, which was very close to the initial fluid bolus of 30 mL/Kg recommended by SSC guidelines [5]. Similarly, in the recent RIFTS trial, authors concluded that there was no mortality benefit using 30 mL/Kg fluid bolus, but in this trial also the amount of fluid used in the patients prior to randomization was more than 30 mL/Kg [6]. Moreover, since the Rivers *et al*. [2] did their randomized controlled trial (RCT) on EGDT in 2001, it broke the taboo of SeS and SS as “Intensive Care Unit (ICU) disease,” and patients got aggressive fluid resuscitation from the very beginning in addition to usual care, which decreased the mortality worldwide [3].

Since the first consensus meeting, the definition of sepsis, severe sepsis, and septic shock had been changed and simplified, and the guidelines regarding screening, initial resuscitation, and target goals had been simplified [7]. The current SSC guidelines do not recommend goal-directed therapy but recommend aggressive fluid resuscitation strategy [8]. SSC guidelines do not have separate guidelines for patients considered at risk for fluid overload like congestive heart failure (CHF), cirrhosis, and end-stage renal disease (ESRD) patients. Due to this, the providers are hesitant to comply with the aggressive fluid resuscitation in such subset of patients. The apparent concern behind the non-compliance is the dangers of fluid overload resulting in cardiac and respiratory failure leading to mechanical ventilation, emergent hemodialysis, and mortality [9, 10].

We conducted this study with the aim to investigate the compliance of ≥30 mL/Kg fluid bolus use among patients who presented with SeS or SS. We hypothesized that aggressively fluid resuscitated patients with CHF do not have higher incidence of complications in comparison to patients without CHF who presented with SeS or SS.

## 2. Methods and methodology

### 2.1. Study design and study population

The study was a single-center, retrospective case-control study that included the patients who presented to the emergency department (ED) of a tertiary care hospital in Fayetteville, North Carolina, United States of America (USA) from January 01, 2017 to December 31, 2019. The study was approved by the Institution Review Board (IRB) of Cape Fear Valley Medical Center (ID: 320–20). The informed consent requirement was waived by the IRB. The charts with discharge diagnosis of sepsis, severe sepsis, and septic shock were retrieved from our institution’s department of medical records. The criteria of severe sepsis and septic shock were based on 1991 and 2001 surviving sepsis campaign definitions [1, 11]. CHF criteria were based on the 2013 AACF/AHA definition [12] (S1 Table in S1 File). Adult patients who presented to the ED with SeS or SS and had a history of CHF were included in the study. Inclusion criteria were: i) patients ≥18 years old, ii) presenting to ED. Exclusion criteria were: i) Age <18 years old, ii) Pregnant, iii) incomplete charts and/or missing information, iv) patients who did not meet the criteria for the criteria of SeS or SS, v) patients who became septic during hospitalization.

The patients with SeS and SS who received ≥30 mL/Kg fluid bolus were divided into case (CHF) groups and control (non-CHF) groups for primary group analysis, and the baseline characteristics and outcomes were compared. The CHF patients were divided into bolus group (BG) if they received ≥30 mL/Kg fluid bolus and no-bolus group (NBG) if they did not receive ≥30 mL/Kg fluid bolus for subgroup analysis.

### 2.2. Study outcomes

#### 2.2.1. The primary outcome

The percentage of patients in both groups who received fluid bolus of ≥30 mL/Kg within 6 hours of presentation.

#### 2.2.2. The secondary outcomes

In-hospital mortality; the total amount of fluid received in 6 hours; time to order antibiotics; intensive care unit (ICU) admission; invasive mechanical ventilation requirement within 24 hours; length of ICU stays, length of mechanical ventilation, needs for vasopressors; source of infection; length of hospital stay; subgroup analysis of CHF patients investigating same outcomes; and the influence of low ejection fraction on fluid bolus compliance.

### 2.3. Data collection

Assuming a 50% variability with the compliance with 30 mL/Kg in patients with SeS or SS, to achieve 95% confidence interval (CI) with ± 5% margin of error, it was estimated that a minimum of 385 charts would be required as a representative sample. The data was extracted from the electronic medical record of the hospital by four internal medicine resident physicians. An extraction form was prepared with the help of Microsoft Excel software, and the extractors were trained on how to use the form and were made familiar with the inclusion and exclusion criteria. The extraction was done in a two-month timeframe (October and November, 2020) using the hospital’s computers, and to ensure uniformity of data extraction two interval meetings were held. The extractors were not made aware of the chart’s group assignment. A randomly selected 70 charts were analyzed by the fifth internal medicine resident physician as the second reviewer.

The time was calculated from the triage in the emergency room (the first recorded time in the triage room as 00:00 hours). The ringer’s lactate and normal saline were considered for the calculation of fluid used for the bolus. CHF diagnosis was based on past medical history (PMH) on the chart, and CHF classification was based on echocardiogram (ECHO) findings within one year but not from the same hospitalization, for example, if the past medical history (PMH) mentioned CHF and no ECHO findings were found within one year then the chart was excluded but if there was no mention of CHF in PMH they were included in the no-CHF group if they met inclusion criteria. The patients who were “do not intubate” (DNI) on the presentation or were made DNI within 24 hours were not included in the calculation of mechanical ventilation rates.

### 2.4. Statistical analysis

Continuous variables were reported as mean (standard deviation) if normally distributed and as median (interquartile range) if not normally distributed. Categorical variables were reported as frequency with percentage. Differences in continuous variables between the groups were evaluated using independent-sample *t-*Test or nonparametric Mann-Whitney U test, as appropriate. Differences in the categorical variables between the groups were analyzed using Chi-square or Fisher’s exact test. A multivariable logistic regression analysis was performed to explore the difference between the groups for in-hospital mortality and mechanical ventilation. A full model was developed to include all the possible predictor variables that could influence the response variable (desired outcome). The predictor variables for the logistic regression model were selected if they had P≤0.25 during univariate analysis or if they had shown to influence the response variable in previous studies regardless of P-value (Model-1). The Variance Inflation Factor (VIF) was analyzed to remove variables that had multicollinearity in the model. To avoid overfitting of the model, the predictor variable to outcome ratio was kept at 10:1 or higher (Model-2). Area Under the Receiver Operating Characteristics Curve (AUC) were compared between the two models. The Model-2 was selected for reporting the results of each outcome to avoid the overfitting. The figures and tables describing the AUC is available in (S1 Table in S1 File). All statistical tests of significance were two-sided and conducted at the 0.05 level of significance. Statistical analyses were performed using STATA 16.1 (Stata Corp, College Station, TX).

## 3. Results

**Fig 1:**
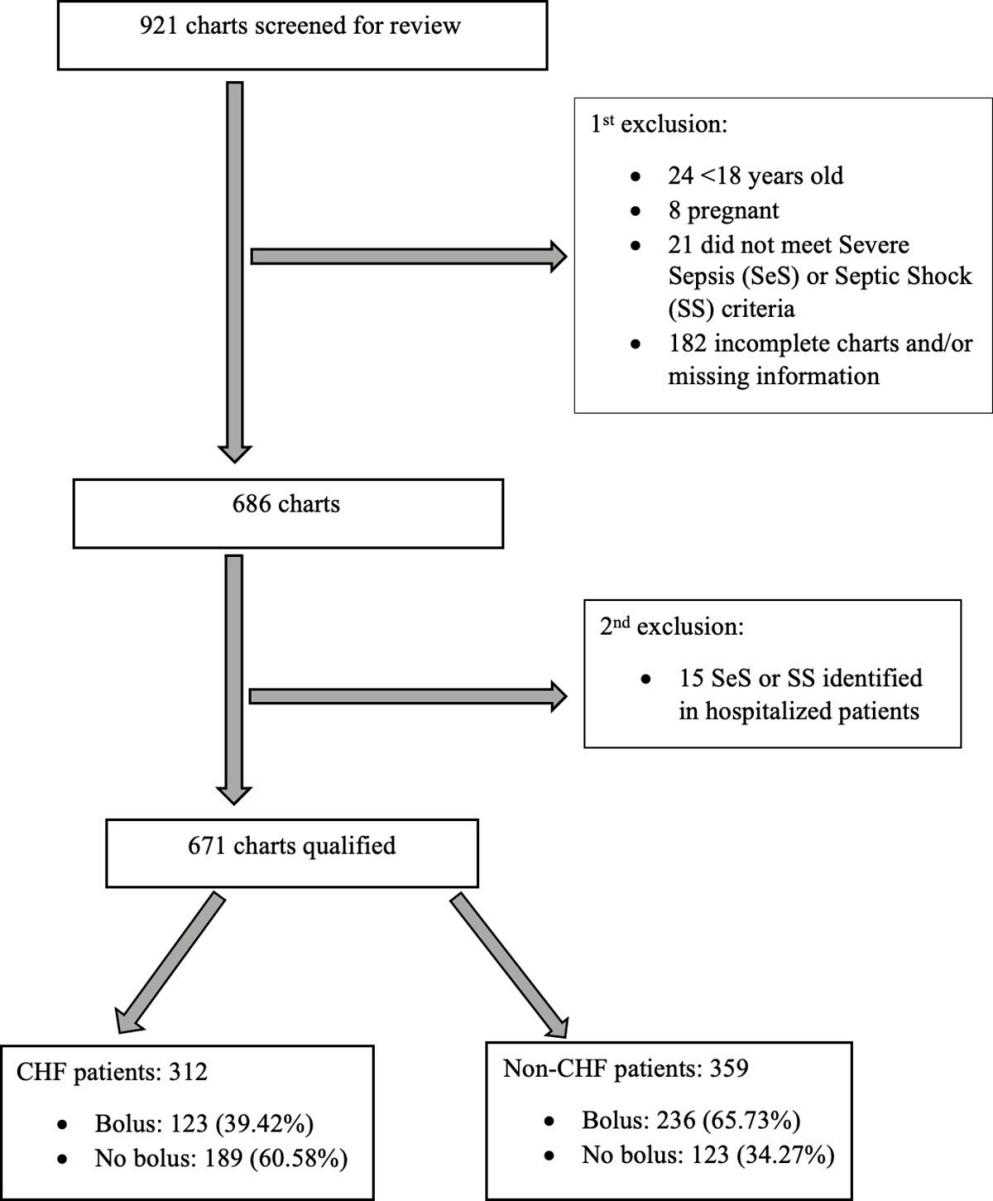
Flow diagram of eligible patient selection. CHF = Congestive Heart Failure.

Initially, a total of 921 patient records were screened for review with the help of “the sepsis and pneumonia coordinator.” Out of the total, 24 charts were <18 years age, 8 charts were pregnant, 21 charts did not meet the criteria for SeS and SS, and 182 charts were either incomplete or missing information. The 15 charts out of the remaining 686 charts were excluded as they were found to have become septic during hospitalization. Total 250 charts were excluded, and the final sample size was 671 (Fig 1).

### 3.1. Primary outcome

Out of 312 patients only 123 received ≥30 mL/Kg fluid bolus in the CHF group, and out of 359 only 236 patients in the non-CHF group received ≥30 mL/Kg fluid bolus [39% vs. 66% (p<0.001)]. Overall, out of 671 patients 359 received ≥30 mL/Kg fluid bolus (54%).

### 3.2. Secondary outcomes

#### 3.2.1. Primary group analysis

This group consisted of the patients who received ≥30 mL/Kg fluid bolus. Patients in the CHF group were relatively older and had more comorbidities as compared to the non-CHF group (Table 1). The total fluid received between CHF and non-CHF groups was almost similar [43.81 mL/Kg vs 44.34 mL/Kg (p = 0.74); 3233.90 mL vs 3070 mL (p = 0.19)]. Mortality was higher in the CHF group [33% vs. 18% (p = 0.002)] as compared to the non-CHF group (Table 2). Univariate analysis revealed higher odds of mortality with the presence of CHF [OR 2.16, 95% CI 1.30–3.57 (p = 0.003)]. Multivariable logistic analysis revealed higher odds of mortality among HFrEF patients [OR 2.70, 95% CI 1.14–6.37 (p = 0.023)]. Each additional 250 mL fluid use decreased the chances of mortality by 12% [OR 0.88, 95% CI 0.82–0.95 (p = 0.002)]. The odds of mechanical ventilation were not increased with the fluid bolus [OR 0.99, 95% CI 0.93–1.05 (p = 0.78)] (Table 3).

**Table 1:** Comparison of baseline characteristics in the primary groups that received ≥30 mL/Kg fluid bolus.

	Case (CHF), n = 123	Control (non-CHF), n = 236	P-value
Age (years), m (SD)	72.28 (12.04)	59.87 (15.68)	**<0.001**
Female, n (%)	59 (47.97)	124 (52.54)	0.67
Ethnicity, n (%)			0.48
White	73 (59.35)	125 (52.97)	
African American	38 (30.89)	87 (36.86)	
Others	12 (9.76)	24 (10.17)	
CCI, m (SD)	6.8 (2.57)	4.3 (3.16)	**<0.001**
ESRD on HD, n (%)	10 (8.20)	15 (6.36)	0.51
Lactic Acid, (mmol/L), m (SD)	3.97 (2.65)	4.36 (3.46)	0.27
SBP (mmHg), m (SD)	86.47 (19.02)	89.91 (17.19)	0.08
DBP (mmHg), m (SD)	52.30 (13.65)	53.49 (12.93)	0.42
MAP (mmHg), m (SD)	63.63 (14.54)	65.55 (13)	0.20
HR (per minute), m (SD)	112.17 (28.95)	115.11 (25.07)	0.31
Temperature (°C), m (SD)	37.12 (1.47)	37.53 (1.42)	**0.01**
RR (per minute), m (SD)	22.1 (7.70)	22.49 (7.58)	0.64
WBC (10^9^/L), m (SD)	14.98 (9.74)	16.04 (11.02)	0.36

m = mean; n = number; SD = Standard Deviation; CCI = Charlson-Comorbidity Index; ESRD on HD = End-Stage Renal Disease on Hemodialysis; SBP = Systolic Blood Pressure; DBP = Diastolic Blood Pressure; MAP = Mean Arterial Pressure; HR = Heart Rate; RR = Respiratory Rate; WBC = White Blood Cell. Significant P-values are in bold.

**Table 2:** Comparison of secondary outcomes in the primary groups that received ≥30 mL/Kg fluid bolus.

	Case (CHF), n = 123	Control (non-CHF), n = 236	P-value
Fluid received (mL/Kg), m (SD)	43.81 (14.34)	44.34 (14.23)	0.74
Fluid in 6 hours (mL), m (SD)	3233.90 (1319.58)	3070 (1006.26)	0.19
Time to order antibiotics (minutes), m (SD)	53.65 (55.73)	65.90 (75.60)	0.11
ICU admission, n (%)	65 (52.85)	118 (50)	0.60
ICU Lo Stay(days), m (SD)	3.35 (3.69)	3.08 (4.49)	0.56
Need for vasopressors, n (%)	83 (67.48)	128 (54.24)	**0.01**
No of vasopressors, n (%)			0.10
1	48 (39.02)	81 (34.32)	
2	24 (19.51)	28 (11.86)	
3	7 (5.69)	10 (4.24)	
4	4 (3.25)	9 (3.81)	
Mechanical ventilation, n (%)	34/104 (32.69) *	48/198 (24.28) *	0.11
Lo mechanical ventilation (days), m (SD)	1.48 (3.47)	1.22 (2.80)	0.43
Source of infection			0.44
Pneumonia	43 (34.96)	74 (31.36)	
UTI	35 (28.46)	62 (26.27)	
Bacteremia	10 (8.13)	25 (10.59)	
Intra-abdominal	18 (14.63)	26 (11.02)	
Skin and Bone	12 (9.76)	28 (11.86)	
Mixed	5 (4.07)	21 (8.90)	
Mortality, n (%)	40 (32.52)	43 (18.22)	**0.002**
Lo Stay (days), m (SD)	12.43 (9.53)	11.55 (16.75)	0.58

M = mean; n = number; SD = Standard Deviation; ICU = Intensive Care Unit; Lo = Length of; UTI = Urinary Tract Infection.

*Mechanical ventilation rate calculated after removing DNI (Do Not Intubate) patients. Significant P-values are in bold.

**Table 3:** Multivariable logistic regression model predicting the outcomes in the primary groups and the CHF subgroups.

	OR	95% CI	P-value
**Mortality in the primary groups** (AUC = 0.852, 95% CI 0.80–0.90)			
Fluid in 6 hours (per 250 mL)	0.88	0.82–0.95	**0.002**
HFrEF	2.70	1.01–5.25	**0.023**
Vasopressors	3.42	2.33–5.01	**<0.001**
Mechanical ventilation	2.75	1.38–5.47	**0.004**
**Mechanical Ventilation in the primary groups** (AUC = 0.780, 95% CI 0.72–0.83)			
Fluid in 6 hours (per 250 mL)	0.99	0.93–1.05	0.78
HFrEF	2.19	0.95–5.06	0.06
Vasopressors	2.49	1.79–3.47	**<0.001**
**Mortality in the subgroups of CHF patients** (AUC = 0.750, 95% CI 0.69–0.81)			
Fluid in 6 hours (per 250 mL)	0.95	0.90–0.99	**0.041**
HFrEF	1.15	0.63–2.09	0.63
Vasopressors	1.32	1.83–3.90	**<0.001**
**Mechanical Ventilation in the subgroups of CHF patients** (AUC = 0.785, 95% CI 0.72–0.84)			
Fluid in 6 hours (per 250 mL)	1.01	0.94–1.03	0.62
HFrEF	1.46	0.76–2.80	0.24
ESRD on HD	2.43	0.96–6.11	0.05
Vasopressors	2.35	1.71–3.22	**<0.001**
**≥30 mL/Kg fluid bolus in the subgroups of CHF patients** (AUC = 0.606, 95% CI 0.54–0.67)			
HFrEF	1.17	0.70–1.97	0.53
DNI	0.75	0.39–1.44	0.39
ESRD on HD	0.86	0.36–2.05	0.75

Congestive Heart Failure = CHF; Heart Failure reduced Ejection Fraction = HFrEF; each vasopressor used in increment = Vasopressors; End-Stage Renal Disease on Hemodialysis = ESRD on HD; Do Not Intubate = DNI. Significant P-values are in bold. AUC represents the Area Under Receiver Operating Characteristics Curve for the final logistic regression model. Full Tables are available in the (S1 Table in S1 File).

#### 3.2.2. Subgroup analysis

This group consisted of CHF patients. The patients in both the bolus and no-bolus groups had a similar distribution in terms of age, sex, and race. HFpEF was the most common type of CHF [59% vs. 61% (p = 0.53)] in both groups (Table 4). The patients in the BG received a significantly higher amount of fluid as compared to the NBG [43.81 mL/Kg vs. 13.46 mL/Kg (p<0.001); 3233.90 mL vs. 1139.85 mL (p<0.001)]. Mortality was almost similar in both groups [33% vs 30% (p = 0.66)] (Table 5). Multivariable logistic analysis revealed each additional 250mL fluid decreased the chances of mortality by 5% [OR 0.95, 95% CI 0.90–0.99, (p = 0.041)]. Also, odds of mechanical ventilation were not increased with the fluid bolus [OR 1.01, 95% CI 0.96–1.06 (p = 0.70)]. The odds of receiving fluid bolus were not influenced by the presence of low ejection [OR 1.17, 95% CI 0.70–1.97 (p = 0.53)], or the DNI status [OR 0.75, 95 CI% 0.39–1.44 (p = 0.39)] (Table 3).

**Table 4:** Comparison of baseline characteristics in the congestive heart failure subgroups.

	Bolus, n = 123	no-Bolus, n = 189	P-value
Age (years), m (SD)	72.28 (12.04)	72. 05 (12.57)	0.87
Female, n (%)	59 (47.97)	78 (41.27)	0.24
Ethnicity, n (%)			**0.03**
White	73 (59.35)	104 (55.03)	
African American	38 (30.89)	78 (41.27)	
Others	12 (9.76)	7 (3.70)	
Ejection Fraction, n (%)			0.53
≥50%	72 (58.54)	116 (61.38)	
41–49%	10 (8.13)	20 (10.58)	
≤40%	41 (33.33)	53 (28.04)	
CCI, m (SD)	6.82 (2.57)	6.97 (2.41)	0.58
ESRD on HD, n (%)	10 (8.13)	16 (8.51)	0.90
Lactic Acid, (mmol/L), m (SD)	3.9 (2.65)	3.45 (2.42)	0.07
SBP (mmHg), m (SD)	86.47 (19.02)	91.53 (12.18)	**0.01**
DBP (mmHg), m (SD)	52.30 (13.65)	54.34 (9.09)	0.11
MAP (mmHg), m (SD)	63.63 (14.54)	66.63 (9.24)	**0.03**
HR (per minute), m (SD)	112.17 (28.95)	103.58 (29.37)	**0.01**
Temperature (°C), m (SD)	37.12 (1.47)	37.18 (1.32)	0.70
RR (per minute), m (SD)	22.1 (7.70)	22.68 (7.26)	0.49
WBC (10^9^/L), m (SD)	14.98 (9.74)	14.47 (7.76)	0.60

m = mean; n = number; SD = Standard Deviation; CCI = Charlson-Comorbidity Index; ESRD on HD = End-Stage Renal Disease on Hemodialysis; SBP = Systolic Blood Pressure; DBP = Diastolic Blood Pressure; MAP = Mean Arterial Pressure; HR = Heart Rate; RR = Respiratory Rate; WBC = White Blood Cell. Significant P-values are in bold.

**Table 5:** Comparison of secondary outcomes in the congestive heart failure subgroups.

	Bolus, n = 123	no-Bolus, n = 189	P-value
Fluid received (mL/Kg), m (SD)	43.81 (14.34)	13.46 (7.70)	**<0.001**
Fluid in 6 hours (mL), m (SD)	3233.90 (1319.58)	1139.85 (750.12)	**<0.001**
Time to order antibiotics (minutes), m (SD)	53.65 (55.73)	51.38 (68.46)	0.78
ICU admission, n (%)	65 (52.85)	81 (42.86)	0.08
ICU Lo Stay (days), m (SD)	3.35 (3.69)	3.08 (2.39)	0.59
Need for vasopressors, n (%)	83 (67.48)	87 (46.27)	**<0.001**
No of vasopressors, n (%)			**0.01**
1	48 (39.02)	49 (26.06)	
2	24 (19.51)	22 (11.70)	
3	7 (5.69)	12 (6.38)	
4	4 (3.25)	4 (2.13)	
Mechanical ventilation, n (%)	34/104 (32.69) *	42/154 (27.27) *	0.34
Lo Mechanical ventilation (days), m (SD)	1.48 (3.47)	1.50 (3.08)	0.96
Source of infection			0.15
Pneumonia	43 (34.96)	67 (35.45)	
UTI	35 (28.46)	50 (26.46)	
Bacteremia	10 (8.13)	34 (17.99)	
Intra-abdominal	18 (14.63)	17 (8.99)	
Skin and Bone	12 (9.76)	16 (8.47)	
Mixed	5 (4.07)	5 (2.65)	
Mortality, n (%)	40 (32.52)	57 (30.16)	0.66
Lo Stay (days), m (SD)	12.43 (9.53)	12.40 (9.77)	0.97

m = mean; n = number; SD = Standard Deviation; ICU = Intensive Care Unit; Lo = Length of; UTI = Urinary Tract Infection.

*Mechanical ventilation rate calculated after removing DNI (Do Not Intubate) patients. Significant P-values are in bold.

## 4. Discussion

In this single-center retrospective study, we found that compliance with ≥30 mL/Kg fluid bolus in the first 6 hours of presentation was low, but the compliance among those with the diagnosis of heart failure was even lower. The mortality and the rate of mechanical ventilation were higher among patients with CHF; however, the use of fluid bolus decreased the chances of in-hospital mortality and did not increase the chances of mechanical ventilation. The chances of receiving fluid bolus were not decreased by the presence of low ejection fraction.

The EGDT trial in 2001 reported benefits of low mortality and less incidence of mechanical ventilation who were assigned to EGDT. The EGDT group had 36.70% of the patients with CHF. Though the subgroup analysis based on CHF was not done, it was a decent representation of patients with heart failure [2]. In the patient-level meta-analysis of trios-trial, the mortality of patients with cardiovascular disease was almost 40% in both EGDT and usual care groups [5]. This high mortality in heart failure patients with severe sepsis and septic shock was consistent with the findings of our study (one-third).

In one of the first studies on CHF and sepsis patients, Oullette and Shah found no increase in hypoxic respiratory failure, mechanical ventilation rate, or mortality with the use of ≥30 mL/Kg fluid bolus in patients with CHF in comparison to patients who did not have CHF. Also, in the same study mortality was significantly associated with failure to comply with sepsis-bundle, with mechanical ventilation, but not with low ejection fraction [13]. Our study found that the need for mechanical ventilation increased the chances of in-hospital mortality. There are a few retrospective studies where the authors concluded that the use of ≥30 mL/Kg fluid bolus in CHF patients was not associated with increased mortality or respiratory failure [14, 15]. Additionally, some studies supported similar findings not only for CHF patients but also for other conditions that are generally considered high risk for fluid overload such as ESRD on hemodialysis (HD) and cirrhosis [16, 17]. Kuttab *et al*. in their study reported that failure to comply with ≥30 mL/Kg fluid bolus within 3 hours of the presentation was associated with increased risk of in-hospital mortality, still the mortality risk was not increased if patients had underlying CHF. Also, the incidence of mechanical ventilation was higher with both the compliant and non-compliant groups, but the risk was not increased by the presence of CHF or ESRD [18]. Our study found that in the patients with CHF presenting with SeS or SS, the use of ≥30 mL/Kg fluid bolus decreased the chances of in-hospital mortality and did not increase the chances of mechanical ventilation, consistent with previous studies. This finding was also reflected in the subgroup analysis.

Abou Dagher *et al*. reported that septic patients with systolic heart failure have higher mortality, rate of mechanical ventilation, use of vasopressor as compared to patients who did not have CHF [19]. In line with this, our study found that HFrEF increased the chances of mortality in SeS and SS as compared to those without CHF. Lemay *et al*. reported that the presence of CHF increased the chances of long-term mortality who survived severe sepsis [20]. Walker *et al*. reported that sepsis was the main non-cardiovascular cause of mortality in CHF patients in the long term, and pneumonia was the primary source of sepsis [21]. Our study also found pneumonia as the primary source of infection. Myocardial depression in sepsis is a known phenomenon with complex pathophysiology [22], but its prognostic implication remains unknown [23]. CHF, similar to old age, diabetes mellitus, and obesity is considered a pro-inflammatory disease condition [24]. The chronic pro-inflammatory state induced by CHF and sepsis-induced acute myocardial depression might explain higher incidences of complications like ICU admission, vasopressor use, and mechanical ventilation even after receiving ≥30 mL/Kg fluid bolus.

The use of vasopressor support, ICU admission was more in EGDT group patients in trio-trial [5]. Jagan *et al*. observed that patients who received ≥30 mL/Kg fluid bolus had a higher incidence of vasopressors use [15]. Zhou D *et al*. found that the combination of more than one vasopressor agents in patients with sepsis who had heart failure was associated with higher short-term and long-term mortality, and a higher incidence of mechanical ventilation [25]. Our study found that the addition of each vasopressor agent increased the chances of mortality and mechanical ventilation in primary and subgroup analysis. In the subgroup analysis of CHF patients, the BG had higher percentages of patients who needed ICU admission and need for vasopressors.

The use of ≥30 mL/Kg fluid bolus in patients presenting with SeS or SS was found to be generally low in the previous studies, regardless of the presence of comorbidities [10, 15, 26]. But the time to initiate fluid resuscitation was longer in patients with heart failure, and in patients with kidney failure, which increased mortality [26]. Also, the CHF patients were less likely to get a total of 30 mL/Kg fluid bolus [15, 18, 26, 27]. The further categorization of CHF based on ejection fraction per AHA guidelines was not done in previous studies. Our study found that the presence of HFrEF did not decrease the chances of getting ≥30 mL/Kg fluid bolus.

Our study had few limitations that were worth mentioning. First, it was a single-center and retrospective study. This limited our understanding of circumstances that led to non-compliance with the fluid bolus. For example, in subgroup analysis, the no-bolus group had better parameters like Systolic Blood Pressure (SBP), Mean Arterial Pressure (MAP), Heart Rate (HR), etc. which could have been the reason for the provider’s decision of not to use ≥30 mL/Kg fluid bolus. Also, there might have been uneven distribution of baseline characteristics between the groups. Though Multivariable logistic regression analysis of outcomes was performed to minimize confounding, residual confounding is possible. We selected the predictor variables after performing univariate analysis and VIF measurement. Hence, we avoided age and included the Charlson-Comorbidity Index (CCI) in our logistic regression models, which may cause consternation among the readers. However, our study had significantly higher number of patients than other similar retrospective studies. The institution provides higher volume of acute care with 142,000 ED visits in 2019 and 120,000 ED visits in 2020, representing a wide catchment area, thus making the results more representative. Second, illness severity scores like APACHE or SOFA score couldn’t be used as many patients were treated outside of the ICU setting, and the necessary data were missing. For the comparison of baseline comorbidity, the CCI was used. Third, the new definitions from 2016 SSC guidelines could not be used because patients from years 2017–2019 were included in the study, and during that time, the institution was transitioning to new sepsis screening criteria, but the majority were screened based on old guidelines. Singer *et al*. mentioned that for patients outside of ICU and suspected sepsis, the predictive validity of SIRS criteria was similar to qSOFA or SOFA score [7]. This makes our study relevant despite the older definitions.

## 5. Conclusion

The utilization of guideline-directed ≥30 mL/Kg fluid bolus in patients presenting with severe sepsis or septic shock within 6 hours of presentation is overall low and even lower among CHF patients. The use of ≥30 mL/Kg fluid bolus in CHF patients presenting with severe sepsis or septic shock seems to confer some protection against in-hospital mortality and is not associated with increased chances of mechanical ventilation. The presence of systolic heart failure does not seem to influence the chance of getting aggressive fluid resuscitation.

## Supporting information

S1 File Full tables explaining study definitions and multivariable logistic regression models. (DOCX)

S1 Data Analyzed data zip file. (ZIP)
