# Supplementary material for: Fluid resuscitation and outcomes in heart failure patients with severe sepsis or septic shock: A retrospective case-control study
Source: PLoS One. 2021 Aug 19;16(8):e0256368. doi: 10.1371/journal.pone.0256368 (PMC8376054; doi:10.1371/journal.pone.0256368)
Supplement: S1 File — (DOCX) [file pone.0256368.s001.docx]

| Severe Sepsis | - two systemic inflammatory response syndrome (SIRS) criteria (temperature > 38 °C (100.4 °F) or < 36 °C (96.8 °F), respiratory rate > 20 breaths/min, heart rate > 90 beats/min or white blood cell count (WBC) >12 × 10 ^9^ /L or < 4 × 10 ^9^ /L, and - sepsis-induced organ dysfunction or tissue hypoperfusion (systolic blood pressure ≤ 90 mmHg or MAP ≤70 mmHg or a fall of >40 mmHg from baseline or serum lactate ≥4 mmol/L regardless of the blood pressure). |
| --- | --- |
| Septic Shock | - sepsis-induced hypotension persisting despite a 30 mL/Kg fluid bolus. |
| HFpEF | - EF ≥50% |
| HFpEF-borderline | - EF =41-49% |
| HFrEF | - EF ≤40% |

Supplementary Table 1. Study Definitions. HFpEF= Heart Failure with preserved Ejection Fraction, HFrEF= Heart Failure with reduced Ejection Fraction, EF= Ejection fraction.

| **Mortality** | **OR** | **95% CI** | **P-value** |
| --- | --- | --- | --- |
| Fluid in 6 hours (per 250 mL) | 0.88 | 0.82 – 0.95 | **0.002** |
| HFpEF | 1.16 | 0.53 – 2.54 | 0.70 |
| HFpEF-borderline | 3.01 | 0.65 – 13.80 | 0.15 |
| HFrEF | 2.70 | 1.01 – 5.25 | **0.023** |
| MAP (mmHg) | 0.99 | 0.97 – 1.02 | 0.86 |
| CCI | 1.13 | 1.02 – 1.26 | **0.014** |
| Vasopressors | 3.42 | 2.33 – 5.01 | **<0.001** |
| Mechanical ventilation | 2.75 | 1.38 – 5.47 | **0.004** |
| ICU admission | 0.44 | 0.20 – 1.13 | **0.038** |

Supplementary Table 2. Multivariable logistic regression model predicting mortality in the primary groups that received ≥30 mL/Kg fluid bolus. Significant P-values are in bold.

MV controlled for: fluid use each 250 mL (Fluid); Heart Failure preserved Ejection Fraction (HFpEF); HFpEF-borderline; Heart Failure reduced Ejection Fraction (HFrEF); Mean Arterial Pressure (MAP); Charlson-Comorbidity Index (CCI); each vasopressor used in increment (Vasopressors); mechanical ventilation; and ICU admission.


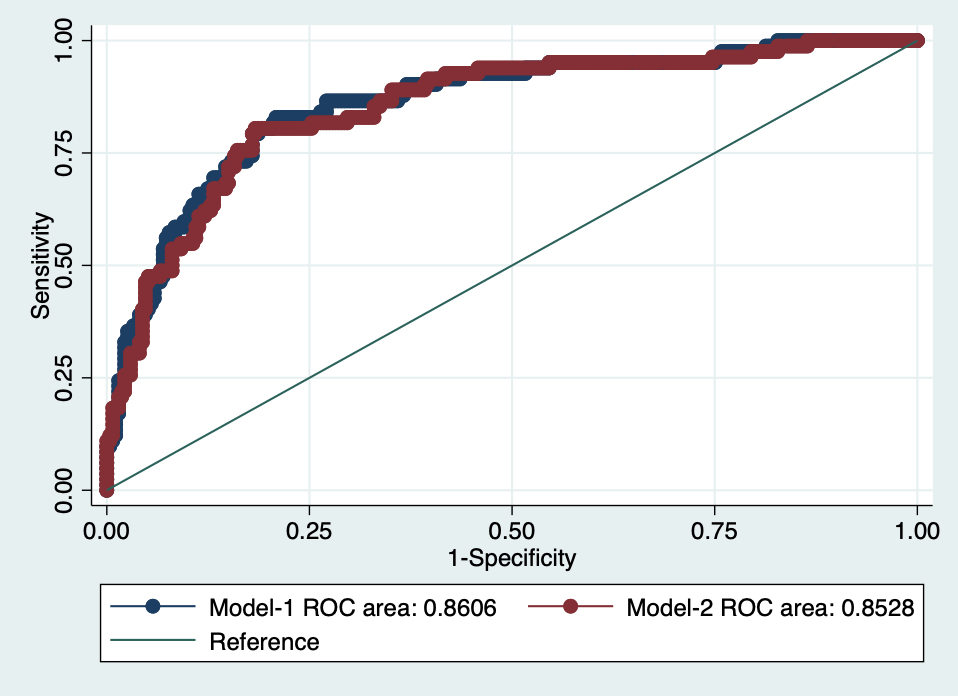


Fig 2. Area Under Receiver Operating Characteristic Curve (AUC) for logistic regression model for mortality in the primary groups. Model-1 95% CI: 0.81 – 0.90, and Model-2 95% CI: 0.80 – 0.90.

| **Mechanical Ventilation** | **OR** | **95% CI** | **P-value** |
| --- | --- | --- | --- |
| Fluid in 6 hours (per 250 mL) | 0.99 | 0.93 – 1.05 | 0.78 |
| HFpEF | 1.34 | 0.61 – 2.90 | 0.45 |
| HFpEF-borderline | 1 | - | - |
| HFrEF | 2.19 | 0.95 – 5.06 | 0.06 |
| MAP (mmHg) | 1.01 | 0.98 – 1.03 | 0.30 |
| CCI | 1.01 | 0.90 – 1.11 | 0.93 |
| ESRD on HD | 1.59 | 0.56 – 4.50 | 0.37 |
| Vasopressors | 2.49 | 1.79 – 3.47 | **<0.001** |

Supplementary Table 3. Multivariable logistic regression model predicting mechanical ventilation in the primary groups that received ≥30 mL/Kg fluid bolus. Significant P-values are in bold.

MV controlled for: fluid use each 250 mL (Fluid); Heart Failure preserved Ejection Fraction (HFpEF); HFpEF-borderline; Heart Failure reduced Ejection Fraction (HFrEF); Mean Arterial Pressure (MAP); Charlson-Comorbidity Index (CCI); End-Stage Renal Disease on Hemodialysis (ESRD on HD); each vasopressor used in increment (Vasopressors).


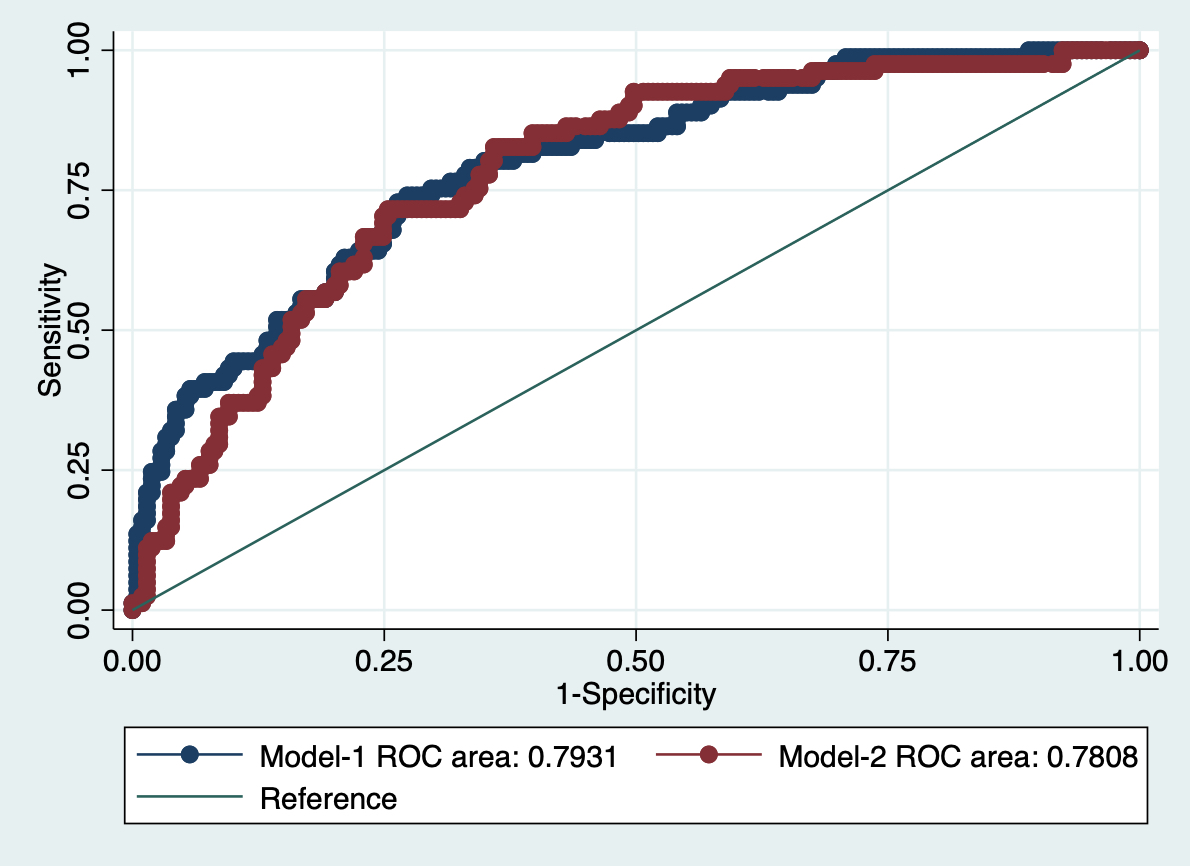


Fig 3. Area Under Receiver Operating Characteristic Curve (AUC) for logistic regression model for mechanical ventilation in the primary groups. Model-1 95% CI: 0.73 – 0.84, and Model-2 95% CI: 0.72 – 0.83.

| **Mortality** | **OR** | **95% CI** | **P-value** |
| --- | --- | --- | --- |
| Fluid in 6 hours (per 250 mL) | 0.95 | 0.90 – 0.99 | **0.041** |
| HFpEF-borderline | 1.94 | 0.80 – 4.67 | 0.13 |
| HFrEF | 1.15 | 0.63 – 2.09 | 0.63 |
| MAP (mmHg) | 0.98 | 0.95 – 1.01 | 0.13 |
| CCI | 1.08 | 0.97 – 1.21 | 0.13 |
| Vasopressors | 1.32 | 1.89 – 3.90 | **<0.001** |
| Mechanical ventilation | 2.72 | 0.67 – 2.60 | 0.40 |
| ICU admission | 0.47 | 0.23 – 0.98 | **0.044** |

Supplementary Table 4. Multivariable logistic regression model predicting mortality in the congestive heart failure subgroups. Significant P-values are in bold.

MV controlled for: fluid use each 250 mL (Fluid); Heart Failure preserved Ejection Fraction-borderline (HFpEF-borderline); Heart Failure reduced Ejection Fraction (HFrEF); Mean Arterial Pressure (MAP); Charlson-Comorbidity Index (CCI); each vasopressor used in increment (Vasopressors); mechanical ventilation; and ICU admission.


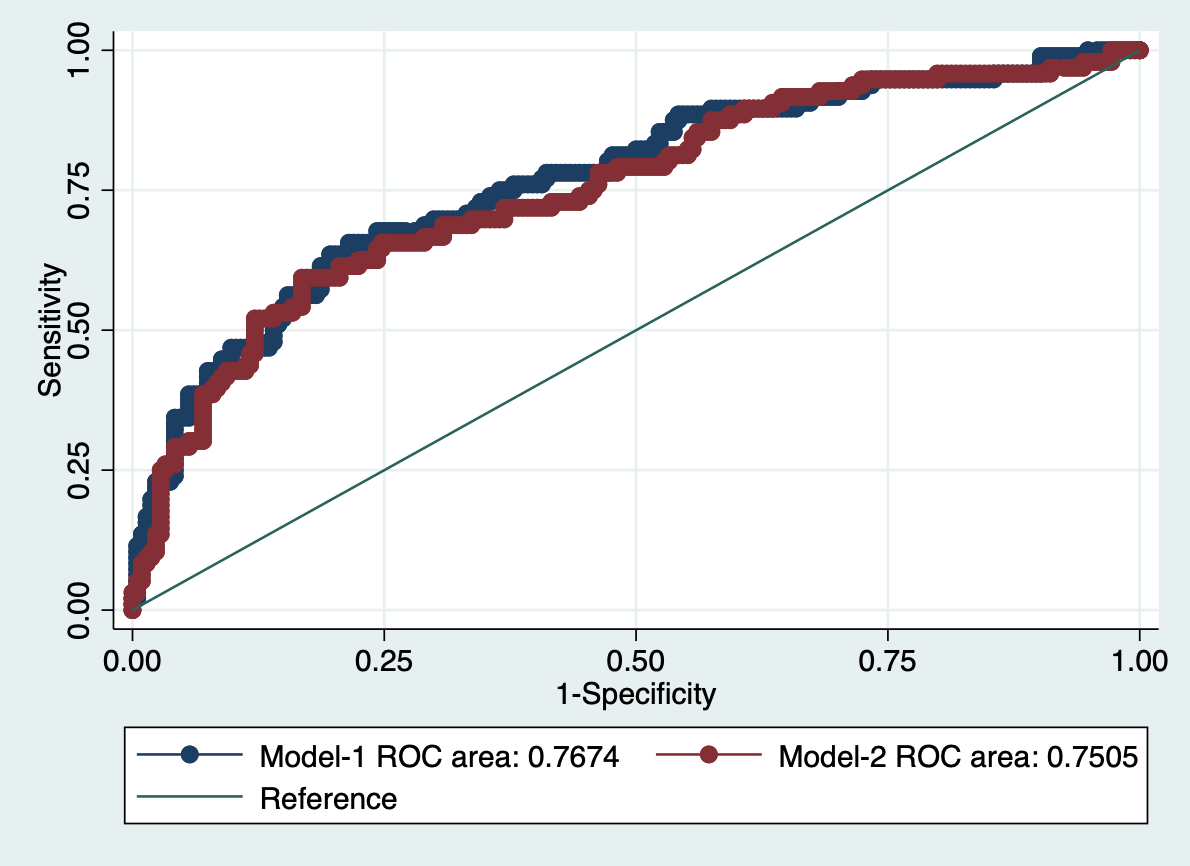


Fig 4. Area Under Receiver Operating Characteristic Curve (AUC) for logistic regression model for mortality in the CHF subgroups. Model-1 95% CI: 0.70 – 0.82, and Model-2 95% CI: 0.69 – 0.81.

| **Mechanical Ventilation** | **OR** | **95% CI** | **P-value** |
| --- | --- | --- | --- |
| Fluid in 6 hours (per 250 mL) | 1.01 | 0.96 – 1.06 | 0.70 |
| HFpEF-borderline | 1.15 | 0.41 – 3.21 | 0.78 |
| HFrEF | 1.46 | 0.76 – 2.80 | 0.24 |
| MAP (mmHg) | 1.01 | 0.98 – 1.03 | .038 |
| CCI | 0.99 | 0.88 – 1.12 | 0.98 |
| ESRD on HD | 2.43 | 0.96 – 6.11 | 0.05 |
| Vasopressors | 2.35 | 1.71 – 3.22 | **<0.001** |

Supplementary Table 5. Multivariable logistic regression model predicting mechanical ventilation in the congestive heart failure subgroups. Significant P-values are in bold.

MV controlled for: fluid use each 250 mL (Fluid); Heart Failure preserved Ejection Fraction-borderline (HFpEF-borderline); Heart Failure reduced Ejection Fraction (HFrEF); Mean Arterial Pressure (MAP); Charlson-Comorbidity Index (CCI); End-Stage Renal Disease on Hemodialysis (ESRD on HD); each vasopressor used in increment (Vasopressors).


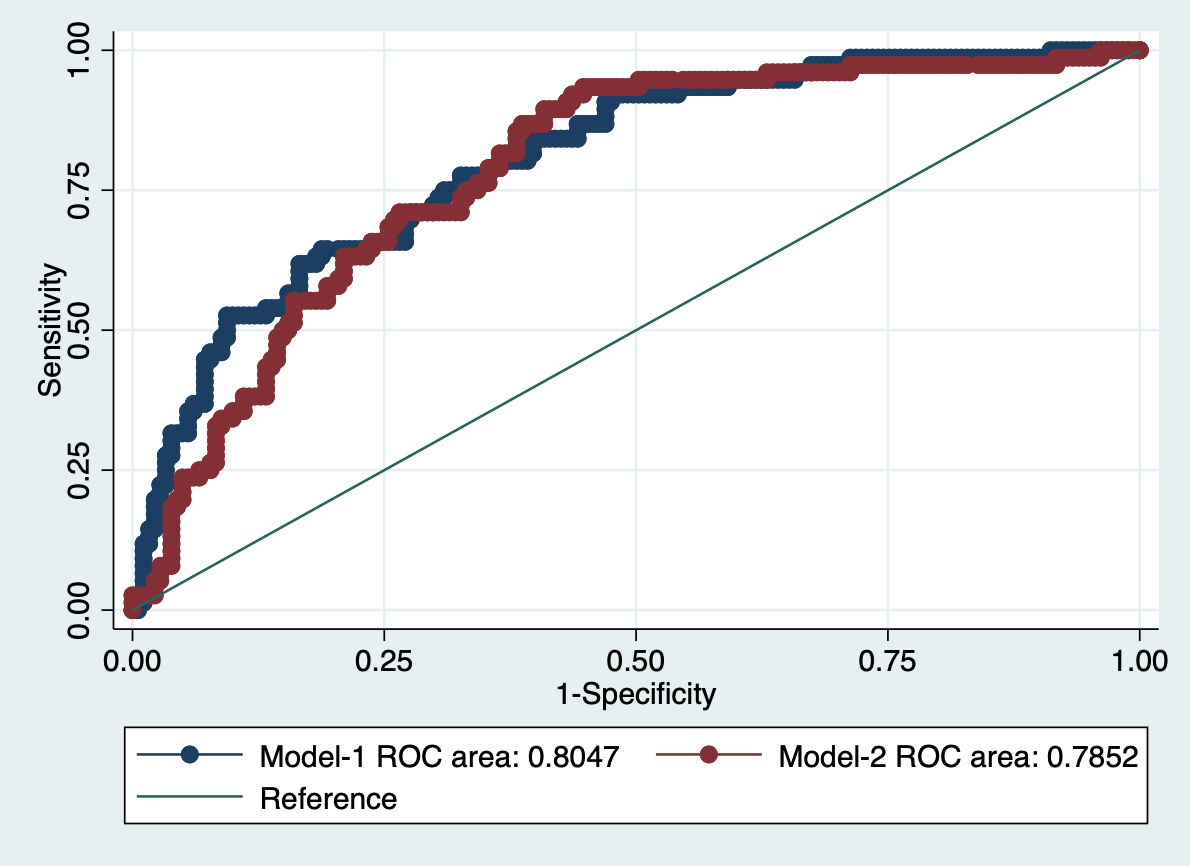


Fig 5. Area Under Receiver Operating Characteristic Curve (AUC) for logistic regression model for mechanical ventilation in the CHF subgroups. Model-1 95% CI: 0.74 – 0.86, and Model-2 95% CI: 0.72 – 0.84.

| **≥30 mL/Kg fluid bolus** | **OR** | **95% CI** | **P-value** |
| --- | --- | --- | --- |
| HFpEF-borderline | 0.76 | 0.33 – 1.74 | 0.52 |
| HFrEF | 1.17 | 0.70 – 1.97 | 0.53 |
| DNI | 0.75 | 0.39 – 1.44 | 0.39 |
| MAP (mmHg) | 0.98 | 0.96 – 1.01 | 0.14 |
| Lactic Acid (mmol/L) | 1.06 | 0.96 – 1.17 | 0.23 |
| CCI | 0.95 | 0.86 – 1.06 | 0.42 |
| ESRD on HD | 0.86 | 0.36 – 2.05 | 0.75 |
| Age (years) | 1.01 | 0.98 – 1.02 | 0.81 |

Supplementary Table 6. Multivariable logistic regression model predicting ≥30 mL/Kg fluid bolus in the congestive heart failure subgroups. Significant P-values are in bold.

MV controlled for: Heart Failure preserved Ejection Fraction-borderline (HFpEF-borderline); Heart Failure reduced Ejection Fraction (HFrEF); Do Not Intubate (DNI); Mean Arterial Pressure (MAP); Lactic Acid (LA); Charlson-Comorbidity Index (CCI); End-Stage Renal Disease on Hemodialysis (ESRD on HD); and Age.


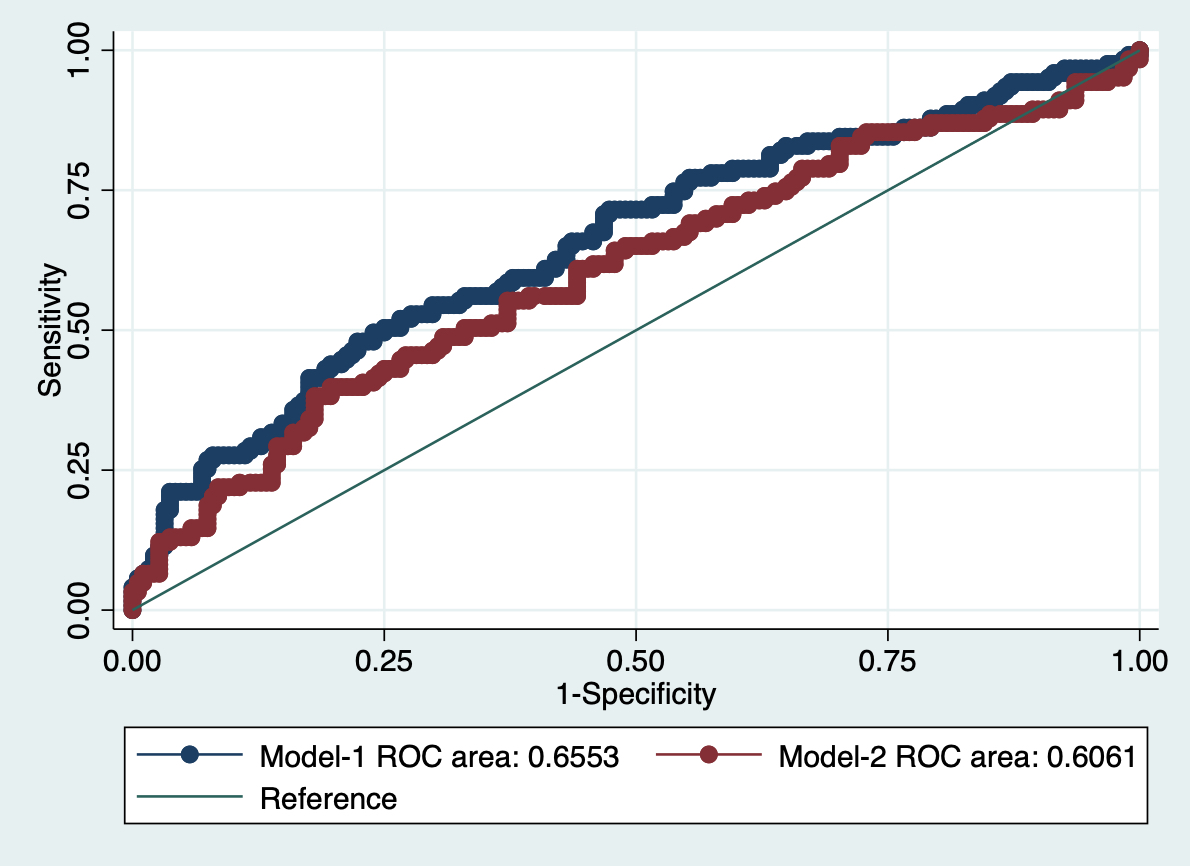


Fig 6. Area Under Receiver Operating Characteristic Curve (AUC) for logistic regression model for ≥30 mL/Kg fluid bolus in the CHF subgroups. Model-1 95% CI: 0.59 – 0.71, and Model-2 95% CI: 0.54 – 0.67.
